# Na^+^/K^+^-ATPase: More than an Electrogenic Pump

**DOI:** 10.3390/ijms25116122

**Published:** 2024-06-01

**Authors:** Ruben G. Contreras, Antonio Torres-Carrillo, Catalina Flores-Maldonado, Liora Shoshani, Arturo Ponce

**Affiliations:** Department of Physiology, Biophysics and Neurosciences, CINVESTAV-IPN, Mexico City 07360, Mexico; gerardo.contreras@cinvestav.mx (R.G.C.); antonio.torres@cinvestav.mx (A.T.-C.); catalina.flores@cinvestav.mx (C.F.-M.); shoshani@cinvestav.mx (L.S.)

**Keywords:** Na^+^/K^+^-ATPase, ion pump, receptor, signalosome, cardiac glycosides, epithelia

## Abstract

The sodium pump, or Na^+^/K^+^-ATPase (NKA), is an essential enzyme found in the plasma membrane of all animal cells. Its primary role is to transport sodium (Na^+^) and potassium (K^+^) ions across the cell membrane, using energy from ATP hydrolysis. This transport creates and maintains an electrochemical gradient, which is crucial for various cellular processes, including cell volume regulation, electrical excitability, and secondary active transport. Although the role of NKA as a pump was discovered and demonstrated several decades ago, it remains the subject of intense research. Current studies aim to delve deeper into several aspects of this molecular entity, such as describing its structure and mode of operation in atomic detail, understanding its molecular and functional diversity, and examining the consequences of its malfunction due to structural alterations. Additionally, researchers are investigating the effects of various substances that amplify or decrease its pumping activity. Beyond its role as a pump, growing evidence indicates that in various cell types, NKA also functions as a receptor for cardiac glycosides like ouabain. This receptor activity triggers the activation of various signaling pathways, producing significant morphological and physiological effects. In this report, we present the results of a comprehensive review of the most outstanding studies of the past five years. We highlight the progress made regarding this new concept of NKA and the various cardiac glycosides that influence it. Furthermore, we emphasize NKA’s role in epithelial physiology, particularly its function as a receptor for cardiac glycosides that trigger intracellular signals regulating cell–cell contacts, proliferation, differentiation, and adhesion. We also analyze the role of NKA β-subunits as cell adhesion molecules in glia and epithelial cells.

## 1. Introduction

For nearly a century, the sodium pump, also known as Na^+^/K^+^-ATPase (NKA), has been extensively studied. This membrane protein complex, identified as EC 7.2.2.13 by the Enzyme Commission [1] or TC 3.A.3.1.1 by the Transporter Classification Database [2], plays a key role in animal cells. It harnesses the chemical energy from ATP hydrolysis to create and maintain an electrochemical membrane potential, which is in turn used in numerous cellular processes, such as the regulation of cell volume, initiating and transmitting action potentials in excitable cells, and facilitating ion and solute transport across epithelial cells [3,4,5].

Recognition of the existence of NKA traces back to the 1930s, when it was observed that a chemical imbalance of Na^+^ and K^+^ ions exists between the intra- and extracellular environments of cells. This imbalance is facilitated by a membrane component reliant on the energy derived from ATP hydrolysis [6,7,8]. In 1941, Dean introduced the term “sodium pump” to describe the uphill energy-dependent transport of K^+^ and Na^+^ in muscle tissue. Skou was the first to describe ion-sensitive ATPase activity in a microsome preparation from crab nerves [9]. Subsequently, the catalytic subunit of NKA, as it is currently known, was isolated and studied [10,11], followed by extensive exploration of its molecular structure and operational mechanism [12,13,14,15,16].

Presently, there is widespread recognition of NKA’s crucial role as a pump, undergoing a repetitive sequence of conformational changes. In each cycle, every functional unit of NKA extrudes three Na^+^ ions and imports two K^+^ ions, utilizing the chemical energy obtained from ATP hydrolysis. This process results in the removal of a net positive charge per cycle, contributing to the establishment of a negative membrane potential, an essential indicator of cellular well-being [17,18,19]. Even before Skou’s functional identification, it had been noted that the enzymatic activity of NKA could be inhibited by ouabain [20,21,22], a plant-derived compound belonging to the group of chemical compounds known as cardiac glycosides (CGs) [23]. Ouabain was initially isolated by Arnaud from the tree *Akantocera ouabaio* [24], and it had been utilized in the treatment of heart conditions [25,26]. Thus, the progress and comprehension of NKA have been closely linked to the understanding of these chemical compounds.

Intriguingly, the influence of CGs on NKA is not only on its pumping capacity, as a growing body of convincing research indicates that ouabain and other CGs may not only inhibit NKA’s pumping function, as, depending on the dosage (either in a nanomolar or micromolar range), it could paradoxically boost its pumping activity directly [27]. Furthermore, CGs have been demonstrated to trigger actions in NKA beyond its pumping function, such as activating various signaling pathways through the transactivation of neighboring proteins, including the non-receptor cytoplasmic tyrosine kinase (SRC), as well as ERK1/2 and PI3K [28,29,30,31].

The growing interest in NKA and its relationship with CGs has been further fueled by the proposition that certain animal species, including humans, naturally produce a variety of endogenous CGs [32,33,34]. Notably, among these compounds, endogenous ouabain has been highlighted [35]. This revelation has led to the idea of classifying these compounds as hormones [36] and sparked the concept of NKA functioning as a hormone receptor involved in modulating diverse physiological processes across various cell types [37,38].

The accumulation of background data has spurred interest in several areas related to NKA. Firstly, there is a focus on achieving a deeper understanding of the operational aspects of the NKA complex as a pump. This involves delving into the intricacies of ion exchange and identifying the sub-components involved in each conformational change. Achieving this objective requires the generation and examination of crystal structures of NKA in different conformational states. Secondly, attention is directed towards investigating the effects of NKA, activated by CGs, on various physiological processes in cells originating from diverse tissues beyond neurons and epithelial cells.

Moreover, emerging evidence indicates that irregularities or changes in its function or composition are linked to several diseases, particularly hypertension and cancer. Hence, substantial research endeavors are presently focused on deciphering and comprehending the complex interplay between NKA and CGs. Furthermore, recent discoveries suggest that apart from CGs, other substances also exert notable influences on NKA, potentially modifying its preference for Na^+^ and K^+^.

## 2. NKA’s Role as an Electrogenic Pump

The fundamental activity of the Na^+^/K^+^-ATPase in pumping Na^+^ and K^+^ ions across the membrane has crucial effects on the physiology of all animal cells. This impact is evident not only in the health of individual cells but also in the physiological functions of entire tissues and organs in metazoan animals.

Arguably, the most essential role of the Na⁺/K⁺-ATPase (NKA) in animal cell physiology is the regulation of cell volume. It is well-known that cells contain various metabolites, causing a natural tendency towards physicochemical equilibrium with water influx. Without a mechanism to continuously expel ions and counteract osmotic imbalances, cells would swell until bursting. The discovery that an energy-dependent Na^+^ transport mechanism is necessary for volume regulation began with descriptions by Harris [39] and Danowski [40] and was further demonstrated by Tosteson and Hoffman [41] through a mathematical model (PLM, the “pump-leak mechanism”) that describes and explains NKA participation in conjunction with passive “leaky” pathways for K^+^ and Cl^−^. (For a more detailed description, see [42]). 

In addition to its primary function as a cell volume regulator, the NKA extrudes three Na^+^ ions and intrudes two K^+^ ions, creating a net outward current. This action leads to hyperpolarization of the cell membrane potential, a fact that, combined with the activity of selective leaky K^+^ channels, stabilizes the membrane potential, which is crucial for excitable cells like neurons, muscle, and cardiac cells [43].

Furthermore, the Na^+^ gradient produced by NKA’s pumping activity serves as a potential energy source that drives the transport, via symporters or antiporters, of various other molecules against their concentration gradients. These include, among many others, the Na^+^/Glucose Symporter, in the intestinal epithelium and renal tubules, the Na^+^/Amino Acid Symporters, which facilitate the uptake of amino acids into the cell, along with Na^+^, the Na^+^/Ca2+ Exchanger, which plays a crucial role in regulating intracellular calcium levels, and the Na^+^/H+ Exchanger, which helps regulate intracellular pH by using the Na^+^ gradient to export H+ ions from the cell, thereby preventing acidification of the intracellular environment [44,45].

**Figure 1:**
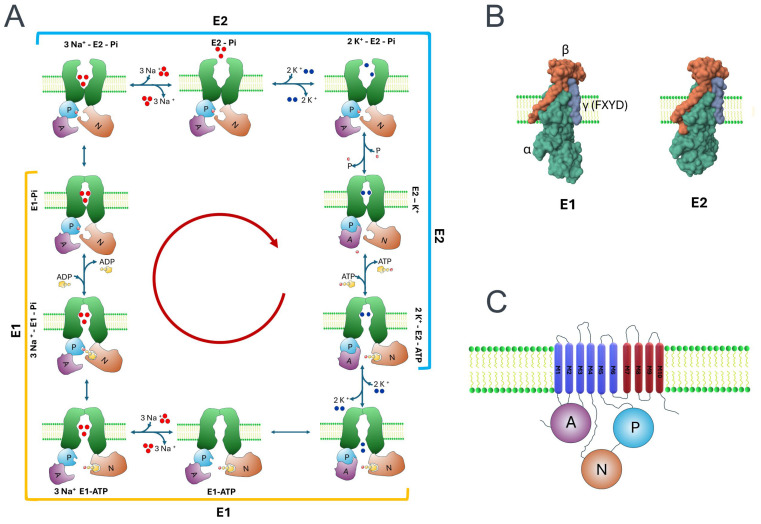
Structure and function of NKA pump. (**A**) The diagram depicts the conformational changes of various domains within the α subunit of NKA, along with the associated ligands for each transition. The red circled arrow denotes the physiological progression of the Post-Alberts cycle. (**B**) Representative crystal images demonstrate structural disparities between the two primary stages, E1 and E2 (created from PB IDs 3WGV and 7WYT [78,79]). (**C**) The topological profile of the α subunit is depicted to highlight its intracellular domains: actuator (A), nucleotide binding (N), and phosphorylation (P) domains.

The pumping function of NKA has been extensively investigated through biochemical, biophysical, molecular, and crystallographic approaches [46,47,48,49]. Initially, biochemical techniques were employed to identify and characterize NKA as an enzyme requiring specific substrates, including Na^+^, K^+^, and Mg2+, as well as ATP, in optimal amounts and proportions for proper functionality [9,18,50,51]. Additionally, it was discovered that the protein responsible for this enzymatic activity transitions between two distinct states, E1 and E2, which can be distinguished through biochemical and biophysical methods [52,53,54]. Albers and Post described the different enzymatic steps and the involved substrates in what is now known as the Post-Albers cycle [55,56,57], which outlines the structural changes executed cyclically by NKA to pump Na^+^ out and K^+^ in [8,13], at a rate of approximately 200 cycles per second [49]. As illustrated in Figure 1A, throughout each cycle, the NKA enzyme alternates between two conformational states, known as E1 and E2. In the E1 state, the enzyme creates an aqueous pathway to the cytoplasmic medium, while in the E2 state, it opens an aqueous pathway to the extracellular medium. In both scenarios, the enzyme adopts an open configuration, facilitating ion exchange, before undergoing occlusion. ATP binding occurs in the E2 state, after the binding of K ions, whereas ATP hydrolysis occurs in the E1 state, following the binding of Na^+^ ions. Physiologically, NKA’s cycle progresses in the sequence indicated, typically in a clockwise direction; however, each stage of the cycle can be reversible, contingent upon substrate availability. It has even been noted that under certain non-physiological conditions, NKA can operate in the opposite direction, potentially acting as an ATP synthase by binding Pi and ADP to generate ATP [58,59].

NKA is classified as a member of the superfamily of P-type ATPases, which transport various compounds vectorially, ranging from H^+^ to phospholipids, at the expense of ATP [60]. Due to its structural and functional characteristics, NKA is categorized as a type IIC-ATPase, sharing close relatives, such as H+/K^+^-ATPase and the sarcoplasmic/endoplasmic reticulum Ca2+-ATPase (SERCA) [61,62,63,64]. Molecular characterization has revealed that each NKA functional unit comprises two or three subunits: α, β, and γ or FXYD. The α subunit performs the catalytic function, the β subunit acts as a chaperone, and the γ or FXYD subunits can modify the transport properties [65,66,67]. Recently, novel functions have been attributed to each subunit, either related to NKA’s role as a pump or as regulators of additional properties. Moreover, NKA in cellular membranes has been described to function as a functional dimer [68,69].

NKA is expressed in animals and in some yeast species [70,71]. Different animal species possess various isoforms of each subunit, with humans and mice having four isoforms of the α subunit, three isoforms of beta, and seven isoforms of FXYD [72,73]. The combinations of subunits vary, with some more abundantly expressed and others limited to certain specific types of tissues [74,75,76,77].

Below is a brief overview of the structure, diversity, and function of these three types of subunits, collectively contributing to NKA’s function as an electrogenic pump. 

### 2.1. α Subunits

The α subunit undergoes cyclic transitions between two conformational states (E1 and E2) to facilitate ion pumping: E1, which opens towards the cytoplasmic side, and E2, which opens towards the external environment. This function relies on the energy derived from ATP hydrolysis, the presence of Mg2+, and the appropriate concentrations of Na^+^ and K^+^ ions. Various studies investigating the structure–function relationship, such as directed mutagenesis and comparative sequence analysis, have identified several distinct domains within the amino acid sequence of the α subunit. Among these domains are motifs that, due to their critical importance, exhibit little variability or remain invariant across the evolutionary diversity of P-type ATPases in general and NKAs in particular [80,81].

According to topological analysis, the α subunit consists of ten transmembrane segments, denoted as M1–M10 (see Figure 1C). The initial six transmembrane segments, M1–M6, fulfill a more direct role in ion passage, alternating as pore and gate, and they are hence termed the core group. The remaining four segments, M7–M10, regulate these properties and are thus designated as the support group. Two intracellular loops, M2–M3 and M4–M5, serve as critical functional domains. The M2–M3 loop, in conjunction with a segment of the amino-terminal region, constitutes a functional domain known as an actuator (A). Meanwhile, the M4–M5 loop, being longer, encompasses both the nucleotide binding (N) and the phosphorylation (P) domains. The A domain functions as a molecular trigger that initiates displacements in the transmembrane segments, thereby causing the pore domain to transition between the E1 and E2 states [74,82]. This domain includes a motif (TGES) implicated in the dephosphorylation step. The N domain serves as an ATP kinase, whereas the P domain contains motifs DKTGT and TGDGVDN. The former includes an aspartate residue (D), which undergoes temporary phosphorylation during catalysis, while the latter is essential for Mg2+ binding [13]. Figure 1B compares the structure of two representative crystals at stages E1 and E2 (images were created in RCSB PDB (http://www.rcsb.org/) [83] with the molecular graphics program Mol* [84] from PDB IDs 3WGV and 7WYT, respectively [78,79]). 

In addition to elucidating the topology of the α subunit, analyses conducted through biochemical, biophysical, and crystallographic investigations have led to the development of a conceptual framework outlining the sequence of events characterizing each cycle of NKA pumping (this process, predominantly executed by the α subunit, is depicted in Figure 1A). Consequently, it is widely recognized that progression through these successive stages necessitates the completion of each preceding step. For example, the occupancy of Na^+^ in their respective binding sites by the α subunit is a prerequisite for its transition to an occluded state and for a structural alteration facilitating the interaction between the N domain and P to facilitate ATP hydrolysis, consequently leading to the phosphorylated state of NKA [13,85]. 

The widely accepted view is that during its pumping cycle, NKA alternately opens an aqueous pathway to both sides of the membrane. This mode of operation contrasts with that of an ion channel, which usually features a single aqueous pathway traversing the membrane. Nonetheless, observations indicate that NKA can, under specific conditions, assume a single aqueous pathway resembling an ion channel, such as, for instance, in the presence of palytoxin [86,87]. Furthermore, mutational changes can transform NKA into an ion channel [88].

Despite the well-documented molecular structure of NKA, the precise molecular mechanism governing the entry and exit of Na^+^ and K^+^ ions within the pump remains elusive. Recent research conducted by Nguyen and colleagues has delved into the intricacies of the cytoplasmic gating mechanism of NKA at the atomic level [89].

### 2.2. β Subunits

β subunits play a crucial role in the structural and functional development of NKA, influencing its transport abilities significantly. Recent research indicates that β subunits may have additional intrinsic functions beyond their connection to NKA’s function. These functions can improve cell adhesion while inhibiting cell motility (reviewed in [67]).

#### 2.2.1. The β Subunit’s Structure and Diversity

The β subunits are transmembrane proteins that typically comprise a single polypeptide chain of roughly 300 amino acids, whose molecular weight ranges from 40 to 60 kDa. Its structure consists of a transmembrane domain (TM) spanning approximately 30 amino acids and an extracellular-facing, globular, immunoglobulin-like domain. Positioned adjacent to the TM7, TM8, and TM9 segments of the α subunit, the TM domain is tilted at an angle of about 40 degrees [52].

Three isoforms of the β subunits are found within the human genome (β1, β2, and β3), each with distinct expression patterns across various organs and tissues. β1 is widely expressed throughout the body, while β2 is predominantly found in the heart, muscles, cartilage, nervous system, and erythrocytes. β3, on the other hand, is expressed in the brain, cartilage, erythrocytes, liver, lungs, retina, and testis [74].

#### 2.2.2. β Subunit Influences NKA Assembly and Pumping Function

β subunits serve as molecular chaperones during the assembly of the NKA complex, aiding in the proper folding and transportation of α subunits to the cell membrane. This chaperone role ensures the accurate assembly and positioning of functional NKA units within the cell membrane [90].

Regarding NKA’s pumping function, β subunits function as regulators of its enzymatic activity, modifying the enzyme’s affinity for Na^+^ and K^+^ ions and influencing its response to diverse regulatory factors, such as hormones and intracellular signaling molecules. Consequently, a crucial function of β subunits is to precisely adjust NKA’s ion transport activity according to cellular needs. Comparative analysis of the biophysical properties of α1β1 and α1β2 reveals changes in Na^+^ affinity, with β2 leading to increased stability of NKA in the Na^+^-occluded E1P state compared to the outward–open E2P state. This alteration arises from a reorientation of the transmembrane domain [91]. 

Several investigations comparing transient currents across various αβ combinations have revealed that both the transmembrane and ectodomain of β subunits play a role in modulating NKA’s transport function by influencing the affinity for K^+^ in a manner not directly associated with the K^+^ binding sites [92].

Furthermore, several investigations suggest that β subunits extend their influence beyond their traditional roles, being implicated in cell adhesion, cell polarity, and the regulation of cell motility.

#### 2.2.3. β Subunit’s Role in Cell–Cell Adhesion

Several recent studies have demonstrated that within epithelial cells, the α₁β₁ heterodimer of NKA, predominantly localizing to the lateral portion of the basolateral membrane domain in mature epithelial monolayers, functions as a cell adhesion molecule [93,94]. This role is primarily attributed to β₁–β₁ interactions among neighboring cells [93,95,96]. Fluorescence resonance energy transfer (FRET) assays have evidenced the proximity of β₁ subunits from adjacent cells, facilitating close interaction [97], which occurs through mutual recognition of β₁ subunits within specific regions, particularly between residues 221 and 229 and 198 and 207, where N-glycans are situated, suggesting a dependence of epithelial junctions on N-glycan-mediated interactions among β₁ subunits of neighboring cells [98]. Furthermore, studies have revealed that alterations in its structure modulate the stability and tightness of intercellular junctions [99]. Beyond this β₁–β₁ interaction, reports indicate an interaction between NKA and E-cadherin, crucial for preserving adherens’ junction integrity, with β₁ subunits playing a significant role in this interaction [100,101]. Additionally, the intensity of β₁–β₁ interaction has been linked to the presence of ouabain and the activation of a signaling pathway in which SRC is involved [102].

In addition to these observations concerning β₁ subunits in epithelial cells, it has been noted that in the retinal pigment epithelium (RPE), where NKA localizes to the apical domain, β2 subunits are essential for targeting NKA units to this domain [103].

In the nervous system, β2 subunits play a role in facilitating the adhesion of glial cells to neurons. Initially identified in glial cells, the β2 isoform, also known as Adhesion Molecule On Glia (AMOG), was found to be involved in cell–cell contacts [104]. Early research indicated that neuro–glial interactions are mediated by the adhesion between the AMOG/β2-subunit and an unidentified partner on the neuron membrane [104,105,106]. More recently, studies on heterologous expression systems have suggested the possibility of a homophilic β2–β2 interaction, although evidence for such interactions within the nervous system is lacking [107]. Based on early findings, Schachner’s group has proposed that astrocytes do not adhere to each other through AMOG/β2 [108], suggesting a potential β2–β2 interaction between astrocytes and cerebellar granular cells. 

### 2.3. FXYD Subunits

The discovery of a third subunit type, now termed FXYD, within the NKA complex, is more recent than that of α and β subunits. In 1978, Forbush et al. first described the presence of a protein component other than β associated with NKA [109]. Nonetheless, it was not until 1997 that Geering’s team provided the initial proof of the presence of a third functional subunit, denoted as γ (now identified as FXYD2), associated with NKA [110]. Subsequent observations revealed various proteins with similar sequences and structures to γ, often exhibiting differential expression in animal tissues [111,112]. In certain mammalian species, including humans, up to seven isoforms are expressed (FXYD1…FXYD7) [113], while in other vertebrate types, like teleost fishes, up to twelve isoforms have been described [114]. Several of these isoforms were discovered and identified in connection with other functions, leading to alternative names besides the FXYD term. FXYD1 is also known as phospholemann (PLM), FXYD2 as γ, FXYD3 as Mat-8 (Mammary tumor marker), FXYD4 as CHIF (channel inducing factor), FXYD5 as dyshaderin or RIC (related to ion channel), and FXYD6 as phosphohippolin [115].

#### 2.3.1. FXYD Diversity and Tissue-Specific Expression

All members of the FXYD family are compact proteins consisting of a single transmembrane segment. The carboxyl-terminal faces the inner side of the membrane, while the amino-terminal faces outward. These proteins are named for a distinctive characteristic, an invariant motif located in the extracellular region. The motif consists of Phenylalanine (F), X (either of Phenylalanine, Histidine, Tyrosine, or Threonine), Tyrosine (Y), and Aspartate (D). Additionally, all FXYD members feature two cysteine residues in the transmembrane region and a serine close to the membrane on the intracellular side [65,116].

FXYD1 is highly expressed in the heart and skeletal muscles [117]. It is also expressed in the smooth muscle, cerebellum, choroid plexus, and liver [118]. FXYD2 is highly expressed in the kidney and pancreas [119]. FXYD3 or Mat-8 is expressed at high levels in the uterus, stomach, and colon and at low levels in the breast, ovary, lung, small intestine, and thymus [120,121]. FXYD4 or CHIF is highly expressed in the kidney [122], and it is also expressed in the salivary glands, stomach, and urinary bladder [123]. FXYD5 or dysadherin is expressed in the lymphocytes, endothelial cells, and basal cells of the stratified squamous epithelium. Notably, it is overexpressed in a variety of cancer cells [124]. FXYD6 is expressed in widely diverse normal tissues and organs and highly expressed in the nervous system [125]. FXYD7 is expressed in the brain [126].

#### 2.3.2. FXYD Functions

FXYD proteins exert tissue-specific control over NKA activity, often through posttranslational modifications, such as phosphorylation (reviewed in [67,111,113]). Several studies indicate that FXYDs influence NKA’s affinity for Na^+^ [49]. Biophysical evidence suggests that FXYD1, FXYD2, and FXYD6 reduce NKA’s apparent affinity for intracellular Na^+^, whereas FXYD4 and FXYD7 enhance it [119]. FXYD7 decreases the affinity for K^+^ ions, thus facilitating enzyme activation under conditions of elevated extracellular K^+^ concentrations [127].

Furthermore, another set of studies delineates the role of FXYD subunits as regulators of NKA activity through various post-translational modifications, including phosphorylation, palmitoylation, and glutathionation [128]. For instance, FXYD1 phospholemman has been identified as a crucial modulator of NKA in the heart through a unique signaling pathway involving post-translational modifications [118].

#### 2.3.3. FXYD Expression as a Biomarker of Cancer Prognosis

In recent years, several studies have linked the expression of different members of the FXYD family to various types of cancer [128,129]. There is even a suggestion that their expression correlates with the prognosis of cancer recurrence. For instance, in colon cancer, the expression of FXYD(2, 3, and 4) has been linked to recurrence prognosis, while FXYD7 is associated with survival prognosis [130]. In Renal Cell Carcinoma, high expression of FXYD3 has been linked to poor prognosis [131]. Glioblastoma exhibits downregulation of FXYD6, suggesting its potential as a biomarker for the diagnosis and prognosis of glioma [125]. Moreover, gene expression analysis indicates FXYD5 overexpression in short-term survivors of high-grade serous ovarian cancer compared to long-term survivors, highlighting its prognostic and predictive significance [132].

### 2.4. Production and Analysis of Crystals Unveil Insights into the Functioning of NKA’s Pumping Mechanism

Enhanced comprehension of the NKA’s components and their functions has been achieved through the generation and analysis of crystal structures representing various conformational states. This approach, initiated in 1977 [133], has progressed significantly, yielding higher-quality crystals suitable for examining the three-dimensional atomic structure of each NKA functional unit’s subunit. These structures also reveal interactions among subunits, as well as with ions and ligands, like Mg2+, ATP, CGs, and lipids. The broader availability of crystals, produced via cryo-electron microscopy or X-ray microscopy [134], capturing different Post-Alberts cycle conformational stages, facilitates the analysis of transition step changes akin to the frames that make up a film. Additionally, the application of docking algorithms has advanced the understanding of how the α, β, and FXYD subunits interact, along with assessing ligand interactions, such as CG and lipids [135].

As shown in Table 1, more than 80 NAK-related crystal structures have been deposited in the Protein Data Bank so far (PDB, https://www.rcsb.org/ [136]). Among these, 41 have been generated within the last 5 years. Specifically, 32 structures were derived from shark (*Squalus acanthias*), 28 from pig (*Sus scrofa*), 10 from human (*Homo sapiens*), 8 from rat (*Rattus norvegicus*), 1 from bovid (*Bos taurus*), 1 from mouse (*Mus musculus*), and 1 from shrimp samples (*Artemia* sp.).

Despite NKA being, fundamentally, an electrogenic pump, as it can operate solely with this cation, most of the crystals developed before 2020 focused on studying the gating and coupling of K^+^ ions and their interaction with CGs (E2-related states), while understanding the E1-related states relied on drawing parallels with the operational mechanism of SERCA. Consequently, it is worth highlighting two significant contributions made in the past two years that have addressed this controversy by creating crystals representing NKA stages linked to the influx of Na^+^ ions from the cytoplasmic side, along with their subsequent ATP occlusion and binding (i.e., E1-related states). 

In their first contribution, Guo and team [137] created and examined cryo-electron microscopy (cryo-EM) structures representing the E1·3Na^+^ and E1·3Na^+^·ATP states with cytosolic gates open and the basic E2·[2K^+^] state, respectively. In this work, which aimed at shedding light on the cytoplasmic Na^+^ entrance pathway and the mechanism of cytoplasmic gate closure coupled with ATP hydrolysis, the authors proposed that a gating mechanism takes place in the cytoplasm in two stages. In the first stage, a gate opens towards the intracellular side during the transition from the E2·[2K^+^] state to the E1·ATP state. Then, two K^+^ are released into the cytosol, while in the second stage, two K^+^ are released into the cytosol. 

In the second contribution, Kanai and coworkers [138] produced two crystal structures of NKA in E1·Mg2+ and E1·3Na^+^ configurations. These structures shed light on the mechanism through which NKA transitions from the K^+^-bound E2·2K^+^ state to an E1 (E1·Mg2+) state, enabling the binding of Na^+^ with strong affinity. This process ultimately results in the closure of the cytoplasmic gate (in E1~P·ADP·3Na^+^) upon the binding of three Na^+^ ions, while concurrently ensuring that the extracellular ion pathway remains sealed [138].

Another remarkable contribution from Kanai’s team has enhanced our understanding of the interaction between CGs and NKA. They obtained and compared crystal structures of the BeF3− complex of NKA, representing the E2P ground state, with eight crystal structures of various CGs, including cardenolides, bufadienolides, and clinically relevant synthetic derivatives, such as rostafuroxin and istaroxime. Their findings revealed that CGs bind to a preformed cavity in NKA without causing significant conformational changes. While CGs with simple steroid cores bind through a “conformational selection” mechanism, those with polar side chains may necessitate helix movements for optimal binding. Furthermore, this study revealed how CGs can stabilize different metal ions in NKA, impacting their inhibitory potency and K^+^ antagonism. Finally, it elucidated how CGs prevent the closure of the K^+^-access channel, thereby influencing their dissociation rate and inhibitory potency [139].

**Table 1:** List of crystal structures of NAK by year and source organism.

PDB ID	Year	Source Organism	Refs.
8K1L	2023	*Artemia salina*	[140]
8JFZ, 8JBK, 8JBL, 8JBM	2023	*Squalus acanthias*	[138]
8JBK	2023	*Sus scrofa*	[138]
7WYS-7WYZ, 7WZ0,	2022	*Squalus acanthias*	[79]
7Y45, 7Y46	2022	*Squalus acanthias*	[79]
7E1Z, 7E20, 7E21	2022	*Homo sapiens*	[137]
7YZR, 7Z04, 7QTV	2022	*Sus scrofa*	[141]
8D3U-8D3Y	2022	*Homo sapiens*	[89]
7X20-7X24	2022	*Rattus norvegicus*	[142]
7D91-7D94, 7DDF-7DDL	2021	*Sus scrofa*	[139]
4XE5	2016	*Bos taurus*	[143]
4RET, 4RES	2015	*Sus scrofa*	[144]
5AVX-5AVZ, 5AW1-5AW3,	2015	*Squalus acanthias*	[145]
5AVQ-5AVW, 5AW4-5AW0			
4HYT	2013	*Sus scrofa*	[146]
4HQJ	2013	*Sus scrofa*	[147]
3WGU, 3WGV	2013	*Sus scrofa*	[78]
3N23	2011	*Sus scrofa*	[148]
2ZXE	2009	*Squalus acanthias*	[149]
3A3Y	2009	*Squalus acanthias*	[150]
2JO1	2007	*Homo sapiens*	[151]
3B8E, 3KDP	2007	*Sus scrofa*	[152]
1XHH	2005	*Sus scrofa*	[153]
1MO7, 1MO8	2003	*Rattus norvegicus*	[154]
1Q3I	2003	*Sus scrofa*	[155]
1BG5	1998	*Rattus norvegicus*	[156]

## 3. Cardiac Glycosides

CGs constitute a category of organic compounds with shared chemical and functional characteristics. Their inherent toxicity has led to their empirical utilization by humans in hunting and warfare endeavors. So far, more than 500 known cardiac glycoside structures have been identified, predominantly originating from plant species that produce them as secondary metabolites. However, a few animal species, including the toad (*Bufo* sp.) and some beetle species, also synthesize CGs as a defense mechanism against predators [157]. A comprehensive examination of the diverse range and distribution of these compounds was conducted by Botelho and coworkers [158].

**Figure 2:**
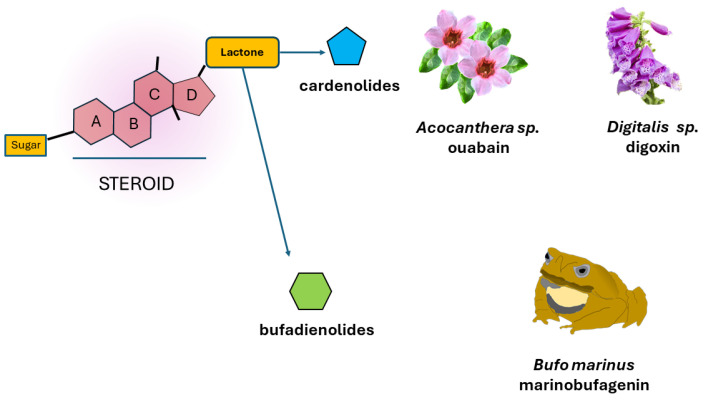
Cardiac glycosides. The figure illustrates the basic chemical structure of CGs, comprising a steroid group with attached sugar and lactone groups, with each containing either five or six carbons. Additionally, it highlights the origins of notable CGs, such as ouabain, digoxin, and marinobufagenin.

One of the primary distinguishing features of CGs lies in their chemical composition (illustrated in Figure 2A), which consists of a C-17 carbon steroid core, known as cyclopentane perhydro-phenanthrene, with a glycosidic moiety located at position C3β, carrying one or multiple sugar units [159,160]. Additionally, there is a lactone ring, either a butenolide or pentadienolide, positioned at C17β, and a hydroxyl group positioned at C14β. CGs are categorized based on the type of lactone group present, with cardenolides containing a pentadienolide and bufadienolides containing a butenolide [161]. The term “bufadienolide” derives from the fact that this type of compound was initially isolated from the toad (*Bufo* sp.), although a wide variety of them have also been identified from plant sources [162].

### 3.1. CG´s Chemical Structure and Activity

Another classical and well-known characteristic of CGs, from which they derive their name, is their capacity to enhance myocardial contractility while also alleviating edema and other symptoms linked to heart failure. This insight was first documented by the English botanist and physician William Withering in the 18th century, who observed these effects through infusions of extracts from the digitalis or foxglove plant (*Digitalis purpurea*). In 1868, French pharmacist Claude Nativelle isolated digitoxin from foxglove extracts [163,164]. Following this, various other CGs were isolated from diverse sources, including ouabain, extracted from plant species of the genus *Acokanthera*, digoxin, derived from species of the genus *Digitalis*, and marinobufagenin, obtained from the skin of toads belonging to the *Bufo* genus, as illustrated in Figure 2. Some of these compounds have been utilized as treatments for heart-related conditions. However, their usage has gradually decreased due to their narrow therapeutic range. Presently, only digoxin and a few other CGs are still utilized in the treatment of heart failure. It was not until the mid-20th century that it was discovered that the effects induced by CGs, whether toxic or therapeutic, resulted from a specific binding with NKA [20,21,22]. 

The therapeutic impact of CGs on the heart stems from their positive inotropic effect, involving NKA and the Na^+^/Ca2+ exchanger (NCX). Partial inhibition of NKA leads to increased intracellular sodium concentration ([Na^+^]i), which reduces NCX activity, resulting in elevated intracellular calcium levels ([Ca2+]i). This cascade induces stronger and more sustained contractions in cardiac cells. Additionally, this mechanism elucidates the ability of CGs to alleviate edema, as NKA inhibition limits Na^+^ absorption, promoting its excretion in urine and enhancing diuresis [165].

An in-depth examination of the crystal structure and functional characteristics of NKA–CG complexes has revealed that cardiotonic glycosides inhibit NKA by binding to the α subunit when it is in an E2 state, characterized by the formation of a cavity facing the extracellular environment. CGs interact with transmembrane segments 1–6 of the α subunit, with the lactone and steroid nucleus coupling in hydrophobic regions, while the glycone group extends outward from the cavity [144]. Once CGs are bound, they compete with K^+^, preventing the closure of the cavity and thus inhibiting the pumping function of NKA.

When examining cardenolides and bufadienolides, differences emerge in their ability to inhibit the catalytic activity of NKA. Cardenolides exhibit greater responsiveness to K^+^ antagonism compared to bufadienolides. Furthermore, it has been noted that cyclization at the C14-C15 position of the steroid ring reduces the affinity for NKA binding [166].

### 3.2. Endogenous Cardiac Glycosides

The observation that there is a correlation between Na^+^ metabolism and peripheral vascular resistance (i.e., hypertension) led to the hypothesis, first suggested by Szent-Györgyi in 1953 and then pursued by Blaustein ([167] and demonstrated by Hamlyn, that human plasma samples contain biologically active inhibitors of NKA [168]. It was further shown that one of them is a steroidal isomer of ouabain (endogenous ouabain, EO) [169], which is secreted by the adrenal cortex under the control of epinephrine and angiotensin II [33]. Further research has identified other endogenous CGs, including digoxin [170], marinobufagenin [34], and telocinobufagin [171].

Endogenous cardiotonic glycosides, classified as steroid hormones of either a cardenolide or a bufadienolide nature, can play a role in regulating blood pressure by affecting cardiac function and Na^+^ handling in the kidney [172]. These compounds unveil an additional function of NKA as a membrane receptor that triggers signaling cascades to regulate cellular processes, such as proliferation, apoptosis, migration, differentiation, and intercellular communication. The diversity of cellular responses is attributed to the various molecules and the different combinations they can form with distinct α, β, and γ subunits.

### 3.3. Novel Cardiac Glycosides and Novel Therapeutic Properties 

A variety of CGs, including both cardenolides and bufadienolides, have recently been shown to possess novel therapeutic properties by influencing NKA. Juventasoside B, a compound isolated from *Streptocaulon juventas*, a plant species used in traditional Chinese medicine, induces NKA to activate SRC and downstream signaling pathways [173]. Calotropin and corotoxigenin 3-O-glucopyranoside (C3OG), both cardenolides extracted from *Asclepias subulata*, a flowering plant native to the deserts of the United States and Mexico, have been shown to hinder the enzymatic activity of NKA and trigger apoptosis [174]. Acovenoside A, a cardenolide extracted from *Acokanthera oppositifolia*, induces apoptotic cell death in non-small-cell lung cancer cells by interfering with EGFR trafficking and inhibiting transcription factor NF-κB activation [175]. Oleandrin, a cardenolide derived from the Nerium oleander plant, has recently gained significant attention for its reported anti-cancer and anti-viral properties [176]. In breast cancer, it has been described to induce immunogenic cell death via the PERK/elF2α/ATF4/CHOP pathway [177]. Super-resolution microscopy studies revealed that treatment of primary cultures of rat kidney with 10 nM of ouabain interfered with the onset of the apoptotic process by inhibiting the activation of the BH3-only protein Bad and its translocation to mitochondria [178].

On the other hand, over the past few years, numerous reports have detailed the discovery of bufadienolides extracted from the skin or venom of various toad species. These compounds have demonstrated noteworthy anti-cancer properties by modulating NKA (reviewed in [179,180,181]); among them, cinobufagin and resibufogenin stand out. Cinobufagin, a cardiotoxic bufanolide steroid secreted by the Asiatic toad *Bufo gargarizans*, has been shown to inhibit proliferation of acute myeloid leukemia cells by repressing c-Myc pathway-associated genes [182]. Resibufogenin, isolated from toad venom and present in Chansu, a traditional Chinese medicine, has attracted growing attention due to its diverse range of pharmacological effects, including effects related to cancer, viral infections, and inflammatory disorders (reviewed in [183,184]). It has been shown to target the α1 subunit of NKA to trigger both the MAPK/ERK pathway and Ca2+-mediated Src/FAK/Paxillin focal adhesion pathways [185].

Within the nervous system, significant findings have emerged regarding CGs. It has been suggested that ouabain may influence nociception by initiating both rapid and gradual signaling in sensory neurons at endogenous levels. The rapid signaling impacts the gating properties of Nav1.8 channels, whereas the gradual signaling leads to downregulation of its expression [186]. In Alzheimer’s disease models, ouabain demonstrates neuroprotective effects by activating a signaling pathway that stimulates transcription factor EB (TFEB), a key regulator of gene transcription within the autophagy–lysosome system, thus reducing tau levels in APP mice [187].

Conversely, numerous studies have outlined the anti-viral properties of CGs, which have been found to exhibit efficacy against various DNA and RNA viral species (reviewed in [188]), including gastroenteritis coronavirus [189], SARS-CoV-2 [190,191], Zika [192], influenza [193], human cytomegalovirus, herpes simplex virus, coronavirus, tick-borne encephalitis (TBE) virus, and the Ebola virus (reviewed in [194,195]).

## 4. NKA’s Role as a Signal-Transducing Receptor

Over two decades ago, the hypothesis emerged that NKA could serve as a signal-transducing receptor for ouabain and other cardiotonic steroids. Since then, numerous studies have detailed the activation of various signaling pathways following the binding of ouabain and other CGs to NKA. These pathways include SRC/EGFR, RAS/MAPK/ERK, IP3K/Akt/mTOR, and related derivatives, such as PLC/PKC. Additionally, it has been observed that this interaction between CGs and NKA induces intracellular calcium oscillations, representing an alternative mode of signaling. The significance of these signaling events has attracted attention due to their involvement in fundamental cellular processes, including the regulation of proliferation, differentiation, apoptosis, and others (reviewed in [196,197,198,199]).

### 4.1. Signaling Pathways Resulting from Sequential Activation of Adjacent Proteins Assembled in Multiprotein Complexes (Signalosomes), Triggered by the Binding of CGs to NKA

**Figure 3:**
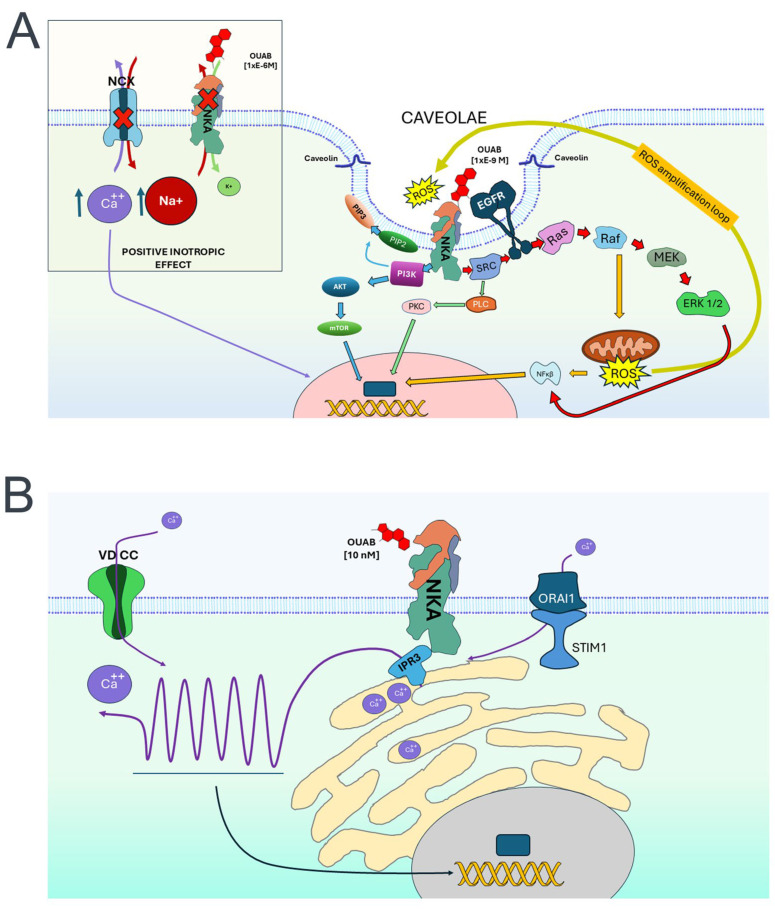
Different signaling pathways activated by the binding of CGs and ROS to NKS. (**A**) The diagram depicts several signaling pathways, including the positive inotropic effect shown in the left inset, as well as those activated by SRC and PI3K transactivation within caveolae. Each signaling pathway is represented by arrows of varying colors. Additionally, the diagram includes the representation of the ROS amplification loop. (**B**) The diagram illustrates the generation of intracellular calcium oscillations and the involvement of molecular components, such as IP3R, ORAi1, and STIM1, along with voltage-dependent calcium channels (VDCCs). Arrows indicate the pathways. The “X” on NCX and NKA indicate diminished activity.

The observation that therapeutic doses of CGs (ouabain and digoxin) induce heart hypertrophy led to the hypothesis that besides inhibiting NKA’s pumping activity, these compounds might facilitate molecular mechanisms associated with cell growth. To evaluate this hypothesis, Xie and Askari assayed the effect of ouabain on cardiac myocytes and found that CGs induce an enhanced expression of *c-fos*, *c-jun*, and the transcription factor AP-1 [200,201]. Closer examination demonstrated that NKA actively contributes to this phenomenon. It also revealed the participation of SRC, which, upon activation by NKA, triggers the activation of EGFR and a Ras/MAPK/ERK pathway [202,203,204]. The fact that ouabain-induced activation of ERK1/2 is partially inhibited by PKC inhibitors led to demonstrating the participation of another pathway including PLC/PKC and intracellular Ca2+ [205]. Additionally, it has been documented that ouabain-induced signaling produces reactive oxygen species (ROS) within mitochondria, following a signaling branch that diverges from the MAPK/ERK pathway originating from Ras [201,206,207,208]. Further research revealed that CGs can activate a PI3K-Akt-mTOR pathway independently of SRC and derived pathways [209,210]. Subsequently, Xie’s team noted two key observations. Firstly, they found that NKA, SRC, and EGFR are co-localized in specific membrane domains. Secondly, they identified two conserved caveolin-binding motifs in the α subunit of NKA. These discoveries prompted them to propose that a subset of NKA units situated in caveolae act as scaffolds and establish interactions with other proteins, including SRC and EGFR, to constitute signaling complexes, also known as signalosomes (Figure 3A). Moreover, it has been discovered that ouabain-induced signaling occurs not only in cardiac cells but also in epithelial cells [196,203,211]. 

Based on the accumulated evidence, Xie’s team extended their hypothesis to suggest the existence of a pre-existing physiological complex where inactive SRC is bound to the α subunit of NKA. They proposed that the binding of CGs disrupts this complex, leading to the activation of SRC.

To delve deeper into the molecular mechanisms, Xie’s team analyzed the amino acid sequence of the α subunit of NKA to identify a 20-amino acid peptide (NaKtide) that disrupts CG-induced activation of SRS-mediated signaling pathways. Additionally, they engineered pNaKtide, a membrane-permeable variant [212]. This experimental progress not only reinforces the hypothesis of NKA transactivation to SRC but also sparks inquiries into the therapeutic promise of this peptide [213].

### 4.2. Signaling Resulted from [Ca2+]i Oscillations Provoked by CGs Binding to NKA

A third signaling pathway triggered by ouabain, causing oscillatory changes of intracellular calcium, and in which NKA also acts as a receptor, was first described by Aperia´s group in epithelial cells [214]. As depicted in Figure 3B, this occurrence revolves around the IP3 receptor (IP3R), which, when activated, releases Ca2+ from the endoplasmic reticulum (ER). Follow-up findings demonstrated that this calcium signaling pathway stimulates cell proliferation and adhesion, as well as protection from apoptosis, resulting from the activation of the nuclear transcription factor NF-κβ. Notably, the activation of IP3R does not stem from the production of IP3 by phospholipase C (PLC) but from a transactivation process from NKA to IP3R, which has been demonstrated to occur colocalized in a protein complex stabilized by ankyrin B [198,215], reviewed in [208]. A corroborating detail indicates that the N-terminal domain of the α subunit of NKA features a motif (LKK) that engages with IP3R [216]. Adding to this signaling process are two additional participants, including the stromal interaction molecule (STIM), which becomes active in response to Ca2+ depletion, subsequently facilitating the gating of calcium release-activated calcium channel protein 1 (Orai1) to replenish ER´s Ca2+ [208].

More recently, a phosphoproteome analysis revealed that in addition to IP3R and STIM1, ouabain induces the activation of a wide variety of calcium and calmodulin-dependent protein kinases (CAMKs), including CAMK type II-γ (CAMK2G), a protein recognized for its role in regulating apoptosis [217].

### 4.3. Signaling Produced after Binding of Reactive Oxygen Species (ROS) to NKA

In addition to the molecular constituents and signaling pathways described above, the involvement of reactive oxygen species (ROS), including superoxide (O_2−_), hydrogen peroxide (H_2_O_2_), and hydroxyl radical (HO−), is noteworthy, as they are both signaling byproducts and initiators, along with CGs, influencing NKA to trigger and sustain diverse pathways. Consequently, in recent years, there has been a noteworthy surge of interest in exploring the related molecular mechanisms and their physiological and pathological effects [207]. The implication of reactive oxygen species (ROS) in the signaling function of NKA was initially observed concurrently with the characterization of NKA as a receptor. As previously mentioned, mitochondria generate ROS because of activation by a Ras-derived pathway stemming from SRC [201,206,207,208]. Later, it was observed that treatment with antioxidants partially abolished ouabain-induced p42/44 mitogen-activated protein kinase (MAPK) activation of NF-kB and protein synthesis, implying that antioxidants themselves elicit effects akin to CGs, affecting both catalytic activity inhibition and signaling [218].

#### Influence of ROS Amplification Loop in Multiple Pathologies

The finding that reactive oxygen species (ROS) instigate NKA-mediated SRC-Ras signaling, coupled with the understanding that ROS are generated in mitochondria as byproducts of SRC-Ras pathway activation, prompted the proposal and subsequent investigation of a positive feedback mechanism. This mechanism, now termed the ROS amplification loop, is believed to continuously operate, resulting in oxidative stress, which is commonly linked to pathological consequences, such as hypertension, obesity, and other related metabolic disorders (reviewed in [207]). There are new descriptions of pathologies in which signaling induced in NKA by oxidative stress plays a pivotal role; these include metabolic diseases, such as hypertension [219], uremic cardiomyopathy [220], obesity [221,222], and nervous-system-related diseases, such as neurodegenerative diseases (encompassing Alzheimer’s disease (AD), Parkinson’s disease (PD), amyotrophic lateral sclerosis (ALS), Huntington’s disease (HD), multiple sclerosis (MS) [223], Obsessive–Compulsive Disorder [224], and bipolar disorder [225]). Furthermore, several reports have detailed how ROS can induce post-translational alterations in NKA, such as carbonylation [226] and S-glutathionylation [227].

With an understanding of both the ROS amplification loop and the inhibitory effects of pNaktide on SRC activation related to NKA, multiple studies have demonstrated that this peptide can inhibit oxidative stress and pathologies resulting from it. For instance, a recent study has revealed that pNaktide regulates metabolic reserve [228] and inhibits obesity [213,222].

### 4.4. Signaling Due to Changes in the [Na^+^]i/[K^+^]i Ratio

Multiple experimental findings suggest that changes in the intracellular sodium and potassium ratio ([Na^+^]i/[K^+^]i) lead to modifications in gene transcription across diverse mammalian cell types, such as neurons, muscle cells, and endothelial cells (reviewed in [229,230]. For instance, it has been observed that elevations in extracellular Na^+^ concentration in human umbilical vein endothelial cells (HUVECs) influence the expression of genes that regulate endothelial function (Fedorov et al. 2021). It has also been recently demonstrated that prolonged incubation (up to 96 h) of HUVEC with CGs (such as ouabain and marinobufagenin) leads to notable increases in the [Na^+^]i/[K^+^]i ratio, accompanied by elevated expression of hundreds of transcripts [231,232]. This and other related evidence in other types of cells have led to the suggestion by Lopina and coworkers of the existence of intracellular sensors of monovalent cations, which they propose could be G-quadruplexes [230], a type of DNA structure that typically forms in guanine-rich regions of genomes [233]. 

## 5. NKA in Epithelial Physiology

Although NKA complexes are now recognized to be widely expressed in animal cells, research has predominantly concentrated on tissues with heightened NKA expression or where its modification exerts the most significant effects, such as the heart or the kidney. 

Epithelial cells play a pivotal role in multicellular organisms by serving as barriers that segregate compartments with distinct fluid compositions, including blood, urine, and gastric fluids. To fulfill these functions, epithelial cells establish connections with neighboring cells through various molecular complexes involved in cell–cell contact, including tight junctions, adherens junctions, gap junctions, and desmosomes. Furthermore, epithelia regulate the movement of ions and solutes between different mediums via a diverse range of membrane transporters, such as pumps, carriers, and ion channels. These transporters exhibit varied distributions across different membrane domains, each characterized by distinct shapes and compositions. Essentially, epithelial cells demonstrate polarization in apical and basolateral domains. In this context, NKA assumes a critical role in generating the electrochemical force required for these processes, whether by establishing a Na^+^ gradient for cotransporters or facilitating ion channels through the creation of a transmembrane potential [44]. Thus, in epithelial cells, NKA is expressed on the basolateral side. Its expression and pumping activity are contingent on the intensity of transepithelial transport and are tightly regulated by various physicochemical factors and organic molecules [234].

### 5.1. MDCK Cells as an Epithelial Model

The availability of cultured cell lines from different animal species, including humans, has facilitated a more profound comprehension of epithelial physiological and structural properties. Among these, MDCK (Madin Darby Canine Kidney) cells, sourced from a dog kidney, likely from the distal convoluted tubule, stand out as one of the most employed [235,236]. Upon seeding, MDCK cells proliferate until reaching confluence, saturating the substratum surface. Subsequently, they organize as epithelial monolayers by forming cell–cell contact structures, such as tight junctions, adherens junctions, desmosomes, and gap junctions. Following this, they polarize their membrane into apical and basolateral domains.

MDCK cells are not only beneficial for examining general epithelial functions but have also been extensively utilized for investigating renal epithelial characteristics [232,233]. In recent years, this cell line also has played a significant role in investigating the interplay between NKA and CGs in epithelial physiology.

### 5.2. Influence of NKA as a Pump on Cell–Cell and Adhesion Contacts

Treating MDCK mature monolayers with ouabain and other CGs (digoxin, digitoxin, palytoxin, oligomycin, strophanthidin, and proscillaridin-A) at doses that inhibit the pump activity of NKA (within the range of 10^−5^ to 10^−6^ M) induces these cells to detach from each other and the substrate without affecting their viability [93,94]. However, following cell detachment, various proteins typically involved in cell–cell contacts and adhesion, including β-catenin and Zonula occludens proteins (ZO-1, and ZO-2), are preferentially located in the nucleus, where they play a role in gene regulation. These observations suggest that NKA, besides its pumping function, also participates in the modulation of adhesion and cell–cell contacts [100,237,238].

### 5.3. NKA Is a Receptor of CGs That Regulates the Epithelial Phenotype

**Figure 4:**
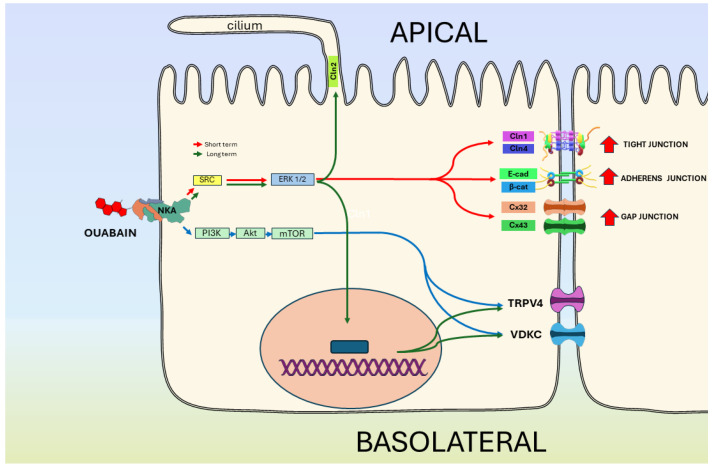
Ouabain–NKA-induced changes in epithelial cultured cells MDCK. The figure outlines the diverse signaling pathways triggered by the binding of ouabain, at nanomolar concentrations, to NKA. It illustrates the resulting effects on cellular components, including voltage-dependent potassium ion channels (VDKC) and TRPV4 channels, as well as the acceleration of ciliogenesis.

Research on MDCK cells has revealed interesting findings regarding the impact of nanomolar doses of ouabain and other CGs (such as digoxin and marinobufagenin). As illustrated in Figure 4, despite not inhibiting the pumping activity of the NKA, these doses induce significant changes in molecular structures involved in intercellular contact and communication. Specifically, when mature epithelial monolayers of MDCK cells are treated with 10 nanomolar ouabain, the following effects on cell–cell contact molecular structures are observed. (1) Increase in the lateral membrane expression of claudins 1 and 4 (Cln1, Cln4), essential components of tight junctions, resulting in reduced paracellular permeability. This indicates a strengthening of cell–cell contact mediated by tight junctions [239,240]. (2) Rise in the expression levels of adherens junction proteins, namely E-cadherin and β-catenin, on the plasma membrane [241]. (3) Enhancement of gap junctional intercellular communication due to the subcellular relocation of gap junction subunits, namely connexins 32 and 43 (Cx32 and Cx43) [239,242].

Moreover, electrophysiological assays (whole-cell patch clamp) demonstrate that in MDCK cells organized as epithelial monolayers, nanomolar doses of ouabain induce the expression of ionic currents attributed to the presence of voltage-gated potassium channels (IKv) [243] alongside ionic currents associated with the presence of TRPV4 channels (ITRPV4) [244].

In addition to the effects observed in cells already organized into a mature epithelial monolayer, it has been noted that the introduction of ouabain into the culture medium during the maturation of epithelial monolayers expedites the establishment of a mature epithelial monolayer and the restoration of polarized membrane domains (apical/basolateral). Consequently, treatment with a concentration of 10 nanomolar ouabain has been observed to hasten ciliogenesis, a critical step in consolidating the epithelial phenotype [245]. Furthermore, cells exhibit a more rapid recovery of ionic current values, attributed to voltage-gated potassium channels [243] and TRPV4 channels [244].

It has also been shown that in all of these effects induced by ouabain, NKA functions as a receptor, initiating various signaling pathways. For cell–cell induced effects, a signaling pathway involving SRC/RAS/RAF/MEK/ERK is implicated, while ion current enhancement involves the PI3K/AKT/mTOR pathway as well.

Furthermore, treatment of mature monolayer MDCK cells with nanomolar doses of ouabain induces changes in various transcripts, including the transcript encoding myosin IXa, which regulates cell shape and collective epithelial cell migration by targeting RhoGAP activity to cell–cell junctions [243]. This finding implicates RhoA and its downstream effector ROCK in the signaling pathways through which ouabain influences cell-to-cell contacts in epithelial cells [244]. These effects collectively underscore the significant role of NKA as a receptor of CGs in modulating the epithelial phenotype.

## 6. Novel Factors Affecting the Activity of NKA

In addition to the well-known influence of CGs on NKA, various chemical agents and environmental stimuli have recently been identified. Thus, it has been reported that hypoxia decreases NKA activity in cardiomyocytes because it promotes a lower expression of the α1 subunit. Inducible hypoxia factor (HIF-1α) is involved in this process [246]. Phytanic acid is a metabolite of chlorophyll-derived diterpenoid phytol [247,248]. Propolis decreases NKA activity [249]. Prion proteins promote the activity of NKA [250]. Rosuvastatin increased NKA protein expression in cultures with primary rat alveolar type II epithelial cells [251]. Anti-cancer agents, plumbagin, and atovaquone inhibit NKA activity by inducing oxidative stress [252]. Oxaliplatin, a compound used to treat cancer of the colon, reduces the activity and expression levels of NKA [253]. Resveratrol, an antidepressant, decreases NKA levels [254]. Prostaglandin E2 upregulates NKA in hepatocytes [255] and downregulates it in epithelial intestinal cells [256]. The aqueous extract of Maca, a *Peruvian herbaceous* plant, enhances the activity of NKA [257]. Lead (Pb) acetate decreases NKA activity in the cerebellum and hippocampus [258]. Bisphenol A impairs renal function by reducing NKA expression [259]. Ecstasy metabolites and monoamine neurotransmitters enhance NKA activity in mouse brain synaptosomes [260]. Inositol pyrophosphate 5-InsP7, a compound that mediates glucose metabolism and insulin secretion from pancreatic β cells, promotes physiological endocytosis and downstream degradation of NKA α1 [261]. Cannabinoids inhibit NKA activity in the nephron [262]. Cholesterol reduces the expression of the α1-isoform but not the α2- or α3-isoform [263]. In gastric epithelia, *Helicobacter pylori* infection causes decreased levels of NKA [264].

## 7. Discussion and Perspective

Because of its function as an electrogenic pump, NKA is a fundamental aspect of the physiology of every animal cell. Consequently, it has driven extensive research, leading to a better understanding of its structure and the mechanisms underlying its crucial role in facilitating the exchange of Na^+^ for K^+^ across cell membranes. Thus, it has been revealed that NKA comprises several subunits: α, β, and γ (or FXDY). Furthermore, it has been elucidated that each of these subunits has a variety of isoforms, which can combine to create different versions of NKA, each possessing distinct biochemical and biophysical properties, many of which remain unidentified. Additionally, it has been observed that subunit isoforms and combinations are expressed differently across various types of tissues with diverse properties. Recent years have witnessed significant progress in comprehending how NKA functions as a pump, which is largely attributable to the growing development of crystals that enable the analysis at the atomic level of the interactions between the amino acids constituting the subunits of NKA and the ions it transports, as well as the ligands that participate in or inhibit its function.

Likewise, the concept of NKA’s role as a receptor triggering a range of signaling pathways that subsequently affect cellular processes has expanded and diversified with new findings. Thus, it is now known that such activity occurs across various cell types beyond heart muscle and epithelia, including neurons [265] and sperm cells [266]. Furthermore, there is a growing acknowledgment of pathological processes involving NKA’s engagement as a receptor, where its interaction with GCs can either instigate or alleviate these conditions. Notably, cancer emerges as a significant focus, with numerous studies underscoring the impact of the GC–NKA interaction on various aspects of cancer progression, such as proliferation [182], adhesion [267], intercellular communication via gap junctions [268], epithelial–mesenchymal transition, and apoptosis [269]. Additionally, other disorders unrelated to cancer but of considerable interest, such as obesity [222], hypertension [270], and metabolic disorders [271], as well as neurological conditions like epilepsy and Alzheimer’s disease [187,272], have been implicated.

Another area of recent interest lies in the relationship between CGs and NKA, as evidenced by the abundance of reports in this article exploring previously uncharted territory regarding CGs. Among these, various bufadienolides stand out, with some having been empirically used in oriental remedies like Chansu and other traditional preparations [184,185]. Additionally, there has been a notable endeavor to elucidate the correlation between the chemical structure of CGs and their effects, both as inhibitors of pump properties and in terms of their signaling effects, as detailed in Section 3.3. Consequently, the effects of CGs derived from natural sources have undergone extensive examination. Moreover, compounds synthesized from natural CGs, including istaroxime and rostafuroxin, among others, have been synthesized and are currently being investigated for their cardio-therapeutic properties or their potential as antagonists to mitigate the effects induced by endogenous ouabain (EO), which contributes to hypertension [273,274].

However, despite the wealth of evidence supporting this signaling phenomenon, it remains paradoxical that a molecular entity primarily tasked with pumping functions can also act as a receptor in circumstances that are not fully understood, thereby sparking controversy and ambiguity [275]. While there is a clearer conceptualization that CGs bind to NKA to inhibit it when it is in the E2 state, supported by various types of evidence, including biophysical and crystallographic data, the understanding of their role in signaling remains obscure. Demonstrations suggest the existence of direct affinity regions between NKA and certain closely associated proteins, such as SRC and IP3R. Nonetheless, it has not been elucidated whether GCs bind to NKA in the same manner to induce signaling as they do when acting as pumping inhibitors. This ambiguity persists because the specificity of binding, as reflected in physicochemical parameters like IC50, does not directly correlate with the signaling pathways they activate.

Thus, after more than sixty years of intensive research, much remains to be discovered about the multifaceted functions of Na^+^/K^+^-ATPase, which has been revealed to be far more than just an electrogenic pump.
